# Archival wild-type poliovirus 1 infected central nervous system tissues of the pre-vaccination era in Switzerland reveal a distinct virus genotype

**DOI:** 10.1007/s00401-023-02545-5

**Published:** 2023-01-31

**Authors:** Nicole Wildi, Stefano Bagatella, Michel C. Koch, Anna Oevermann, Torsten Seuberlich

**Affiliations:** Division of Neurological Sciences, Vetsuisse Faculty, University of Bern, Bern, Switzerland

Poliovirus 1 (PV1), a member of the species *Enterovirus C*, causes poliomyelitis and has almost been eradicated globally following world-wide vaccination campaigns since the 1960s. Yet, rare fatal cases due to infections with wild-type PV1 or circulating vaccine-derived poliovirus are still being reported in some developing countries [6, 7]. PV1 is among the bests studied RNA viruses; however, PV1 genomes have been sequenced mostly from feces samples or after passage in cell culture and to our knowledge never from nervous tissue directly. Thus, we know little about the genetic diversity of neurovirulent wild-type strains in the CNS of affected patients in the pre-vaccination era.

Through archive mining, we retrieved paraffin-embedded autopsy brain samples and related medical records of a neonate (patient #1) and an adult (patient #2) diagnosed with poliomyelitis from the early 1950s, before poliovirus vaccines were available (Table 1). Both patients presented progressive neurological signs and died despite attempts of negative pressure ventilation. The autopsy tissues had been submitted to our division by a hospital in Switzerland for neuropathological assessment and showed a severe polioencephalomyelitis (patient #1), and poliomyelitis (patient #2) (Table 1). The lesions were more severe in the neonate than in the adult, and there were differences in the histopathological lesion pattern, which were consistent with reports in the literature [1, 5]. Lesions in patient #1 consisted of a marked infiltration of phagocytotic cells (microglia, macrophages, and neutrophils), while lesions in patient#2 involved almost exclusively microglia/macrophages (Fig. 1a–e, Supplementary Fig. 1 a–f and Supplementary Table 2—online resources).

**Table 1:** Summary of findings in two patients with poliomyelitis

Case	Age, year of death	Tissue availability	Inflammatory lesions	Viral RNA (ISH)	Poliovirus 1 strain (scaffold length/kmer coverage)
Patient #1	15 days, 1954	Spinal cord	+	+	Poliovirus 1 CHE2132/54 (7442 nt/480)
		Medulla oblongata	+	+	
		Pons	+	+	
		Thalamus	+	−	
		Cortex	+	−	
Patient #2	26 years, 1953	Spinal cord	+	+	Poliovirus 1 CHE1884/53 (7019 nt/53)

*ISH* in situ hybridization, *nt* nucleotides, + present; − absent

**Fig. 1:**
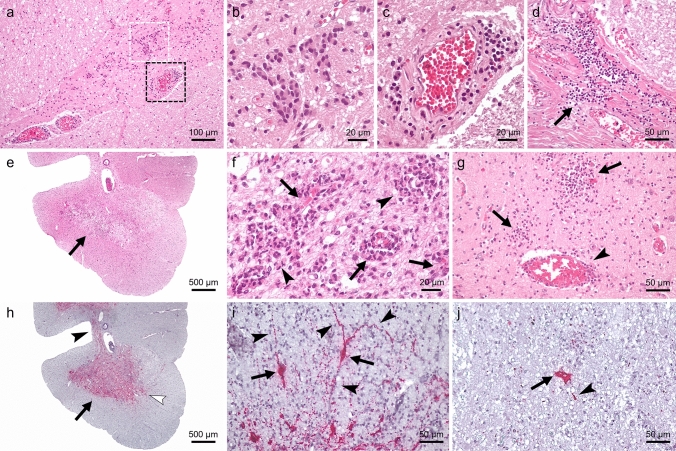
Histopathological findings and localization of Poliovirus 1 RNA in archival brain tissues of patients with poliomyelitis. In patient #2, the anterior horn of the spinal cord (**a**) shows glial nodules (white square, **b**) and perivascular cuffs composed of microglia/macrophages (black square, **c**). Additionally, moderate lymphocytic meningitis is observed (**d**, arrow). In patient #1, the anterior horn of the spinal cord is severely affected (**e**) and displays perivascular cuffs (arrows) and intraparenchymal clusters (arrowheads) composed of microglia/macrophages or neutrophils. Similar clusters are also found in the motor nucleus of the trigeminal nerve in the pons (**g**, arrows), accompanied by thin mononuclear perivascular cuffs (**g**, arrowhead). ISH reveals clear viral targeting of the spinal cord grey matter (**h**), as well as the grey commissure (**h**, black arrowhead) and, partially, the posterior horn (**h**, white arrowhead). Viral RNA is present in neuronal cell bodies (**i**–**j**, arrows) and processes (**i**–**j**, arrowheads) in the spinal cord of patient #1 (**i**) and #2 (**j**). Magnification: **a** = ×10; **b**, **c**, **f** = ×40; **d**, **g**, **i**, **j** = ×20; **e**, **h** = ×2. ISH negative control, additional histopathological lesions, and immunohistochemical staining are shown in Supplementary Fig. 1 and the immunolabeling distribution in Supplementary Table 2 (online resource)

To demonstrate infection with PV1 and to obtain genetic virus information, we extracted RNA from CNS tissues of both patients and submitted it to high-throughput sequencing followed by bioinformatics analysis (Supplementary methods—online resource). We obtained two, coding-complete PV1 genome sequences, which we designated strains CHE2132/54 (patient #1; GenBank OP828753.1) and CHE1884/53 (patient #2; GenBank OP828752.1).

Based on the new PV1 sequences, we designed a probe for RNA in situ hybridization (Supplementary methods—online resource), and demonstrated viral genomic RNA within CNS tissue in both patients (Fig. 1h–j), confirming that they were affected by PV1 poliomyelitis.

For strain CHE2132/54, only 3 nucleotides (nt), and for CHE1884/53, 432 nt are missing at the genome 5′ end in the untranslated region (UTR) when compared to the PV1 reference strain (Mahoney; GenBank V01148.1).

Both strains showed a sequence similarity of 91% to each other but less than 83% compared to other PV1 strains (Supplementary Table 3—online resource). Phylogenetic analysis corroborates this finding and shows that both strains cluster together but are clearly distinct from previously reported PV1 strains (Fig. 2). Conversely, the encoded polyprotein sequences are highly conserved with > 97% identity compared to other PV1 strains, indicating a high selection pressure (Supplementary Table 3—online resource). Of particular note is that both new sequences showed genomic key features of neurovirulent PV1 strains, e.g., an adenine residue at position 480 of the 5′-untranslated region (UTR), which contribute to neurovirulence in the Mahoney strain [3] (Supplementary Fig. 3—online resource).

**Fig. 2:**
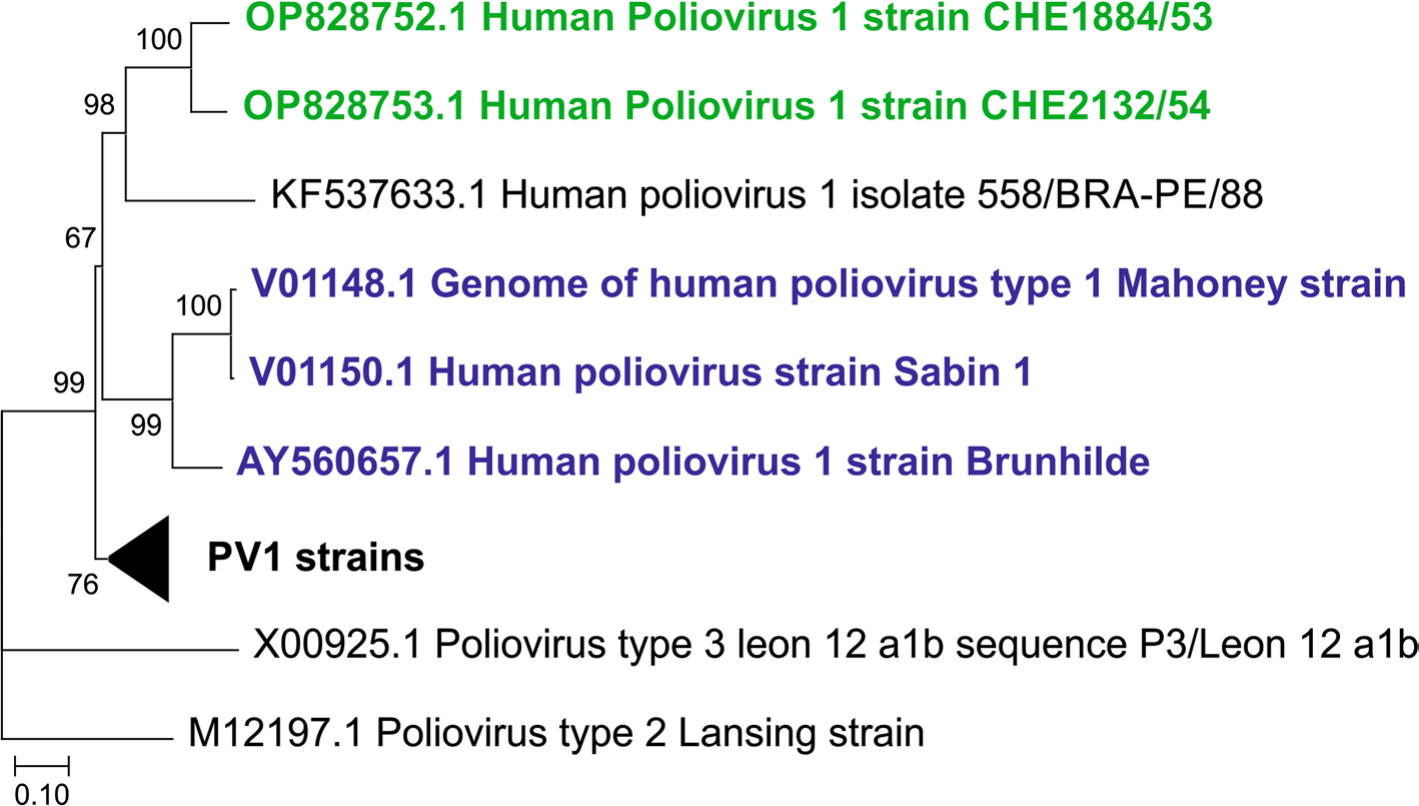
Phylogenetic comparison of archival Swiss poliovirus with full-length genomes of wild-type polioviruses. Available wild-type PV1 genome sequences from sewage/feces specimen (*n* = 18) are closely related (compressed triangle). However, the Swiss archival strains CH1884/53 and CH2132/54 (green) are closely related only to a PV1 strain reported from Brazil (KF537633). The third branch represents the strains used for oral vaccine development (blue). Alignment was performed using MAFFT [2] and the tree was constructed using MEGA X [4]. The complete tree is shown in Supplementary Fig. 2 (online resource)

In conclusion, by sequencing almost full genomes of PV1 in 70-year-old archived paraffin-embedded CNS tissues, we found substantial genetic divergence from previously described PV1 strain genomes. Whether this divergence reflects viral adaptions to the nervous tissue or indicates particular PV1 strains that circulated in the middle of the twentieth century in Europe will need to be subject of continued research.

## Supplementary Information

Below is the link to the electronic supplementary material.

Supplementary file1 (DOCX 6225 kb)

## Data Availability

High-throughput sequencing data are available at the sequence read archive of the national center for biotechnology information, Bioproject PRJNA899910 (https://www.ncbi.nlm.nih.gov/sra/PRJNA899910). Poliovirus 1 sequences are available at ncbi GenBank with accession number OP828752 (patient #2) and OP828753 (patient #1).
