# Supplementary material for: Archival wild-type poliovirus 1 infected central nervous system tissues of the pre-vaccination era in Switzerland reveal a distinct virus genotype
Source: Acta Neuropathol. 2023 Jan 31;145(3):357–9. doi: 10.1007/s00401-023-02545-5 (PMC9888746; doi:10.1007/s00401-023-02545-5)
Supplement: Supplementary file 1 — Supplementary file1 (DOCX 6225 kb) [file 401_2023_2545_MOESM1_ESM.docx]

**Supplementary information**

**Materials and Methods**

**High-throughput sequencing and bioinformatics**

Three 10-µm thick FFPE slices were taken from each available CNS region (Table 1) and deparaffinized with xylene, followed by RNA extraction using the RNeasy FFPE kit (Qiagen) according to the manufacturer's instructions. RNA extracts were quality controlled, pooled per patient, submitted to HTS library preparation (Corall total RNA-Seq kit, Lexogen) and single-end sequenced with 100 cycles on an Illumina NovaSeq 6000 machine. In total ~78 Mio reads were generated for the extracts of patient #1, and ~69 Mio reads for the extracts of patient #2. Reads were quality-controlled (fastqc vers. 0.11.7; https://github.com/s-andrews/FastQC), trimmed (fastp 0.12.5; [3]), and host-derived sequences were removed by aligning reads to the human reference genome (assembly GRCh38.p14) using STAR v. 2.7.3 [4]. Non-aligned reads were assembled with SPAdes v.3.12.0 [15]. The resulting contigs were screened for homologies on the nucleotide and amino acid levels using BLASTn v. 2.10.1+ [2] against viral nucleotide and protein sequences in GenBank (https://www.ncbi.nlm.nih.gov/genbank/) and DIAMOND v. 2.09 [1] against viral protein sequences in UniProt (https://www.uniprot.org/), respectively. (Databases were downloaded on 22 June 2021.).

**In situ hybridization**

To visualize viral RNA in situ, we used the RNAscope® 2.5 HD Assay-RED (acdbio) with a customized RNA probe (target region nt 2196 - 3274 of Poliovirus 1 strain CHE2132/54 genome, catalogue number 1135551-C1) according to the manufacturer's instructions. All available regions (Table 1) were mounted on glass slides and tested for the presence of Poliovirus 1 RNA. Brain tissues from patient without inflammatory lesions was used as a negative control. As a technical positive control, brain sections of cattle with bovine astrovirus CH13 infection were used along with a bovine astrovirus specific RNAscope probe [11].

**Phylogenetic analysis**

Coding complete sequences of both PV1 strains identified in this study and full-genome poliovirus sequences available from GenBank were aligned with MAFFT vers. 7.475 [9] and trees were built with iqtree vers. 2.0.3 [14] with 1000 bootstraps. The resulting trees were visualized and manually curated with MEGA X [12].

**Immunohistochemistry staining for CD3, CD20, Iba1 and neutrophil elastase**

Slides were deparaffinized by three xylol washes (3x5min) followed by 3 min in 100% ethanol. Endogenous peroxidase was blocked with 3% H2O2 diluted in methanol for 15 min. Antibodies and target retrieval used are listed in Supplementary Table 1. Biotinylated Goat anti-Mouse & Rabbit IgG (H+L) (Ready to Use) (abcam) was used as secondary antibody in combination with streptavidin-biotin immunoenzymatic antigen detection system (abcam).

**Tables**

**Supplementary Table 1:** Protocols for different IHC techniques

|  | **Anti-CD3**  **(Dako)** | **Anti-CD20**  **(Dako)** | **Anti-Iba1**  **(Wako)** | **Anti-human neutrophil elastase**  **(Hycult Biotech Inc.)** |
| --- | --- | --- | --- | --- |
| **Target retrieval** | none | none | Citrate buffer pH 6.0  Heated in microwave at 95° for 15 minutes | none |
| **Antibody** | 1:600; overnight at 4°C | 1:100; 1h at RT | 1:500; 1h at RT | 1:50; 1.5h at RT |

**Supplementary Table 2:** Immunolabeling distribution of immune cells. ++ = abundant; + = moderate; +/- = rare. The IHC staining were performed on tissue with the most severe lesions of each patient and where viral RNA was found.

| **Case** | **Brain regions** | **Neutrophils**  **(Neutrophil elastase)** | **Microglia/Macrophages**  **(Iba1)** | **T-Lymphocytes**  **(CD3)** | **B-Lymphocytes**  **(CD20)** |
| --- | --- | --- | --- | --- | --- |
| **Patient #1** | Spinal cord | ++: parenchymal clusters, perivascular cuffs, and meninges | ++: parenchymal clusters, perivascular cuffs, and meninges | \| +/-: randomly scattered in the parenchyma \| \| --- \|   none | +/-: scattered in the meninges |
|  | Medulla oblongata | ++: parenchymal clusters; + perivascular cuffs and scattered in the parenchyma | ++: parenchymal clusters, perivascular cuffs, and meninges | \| -- \| \| --- \|   none | +/-: scattered in the parenchyma (often at the periphery of parenchymal clusters) and perivascular cuffs |
|  | Pons | +: parenchymal clusters; +/- perivascular cuffs | ++: parenchymal clusters, perivascular cuffs | +/-: scattered in the parenchyma and in perivascular cuffs | +: scattered in the parenchyma (often at the periphery of parenchymal clusters) and perivascular cuffs |
| **Patient #2** | Spinal cord | +/-: scattered in the parenchyma | ++: parenchymal clusters, perivascular cuffs, and meninges | +/-: scattered in the parenchyma | +/-: scattered in the parenchyma |

**Supplementary Table 3:** Percentage of nucleotide (a) and amino acid (b) identity of the different PV1 strains. Our strains (green) are similar to each other, but they differ from the other PV1 strains, including the Brazilian strain (KF537633). The alignment was performed using MAFFT [9]. The graphics are assembled using CorelDraw X6 Version 16.0.0.707


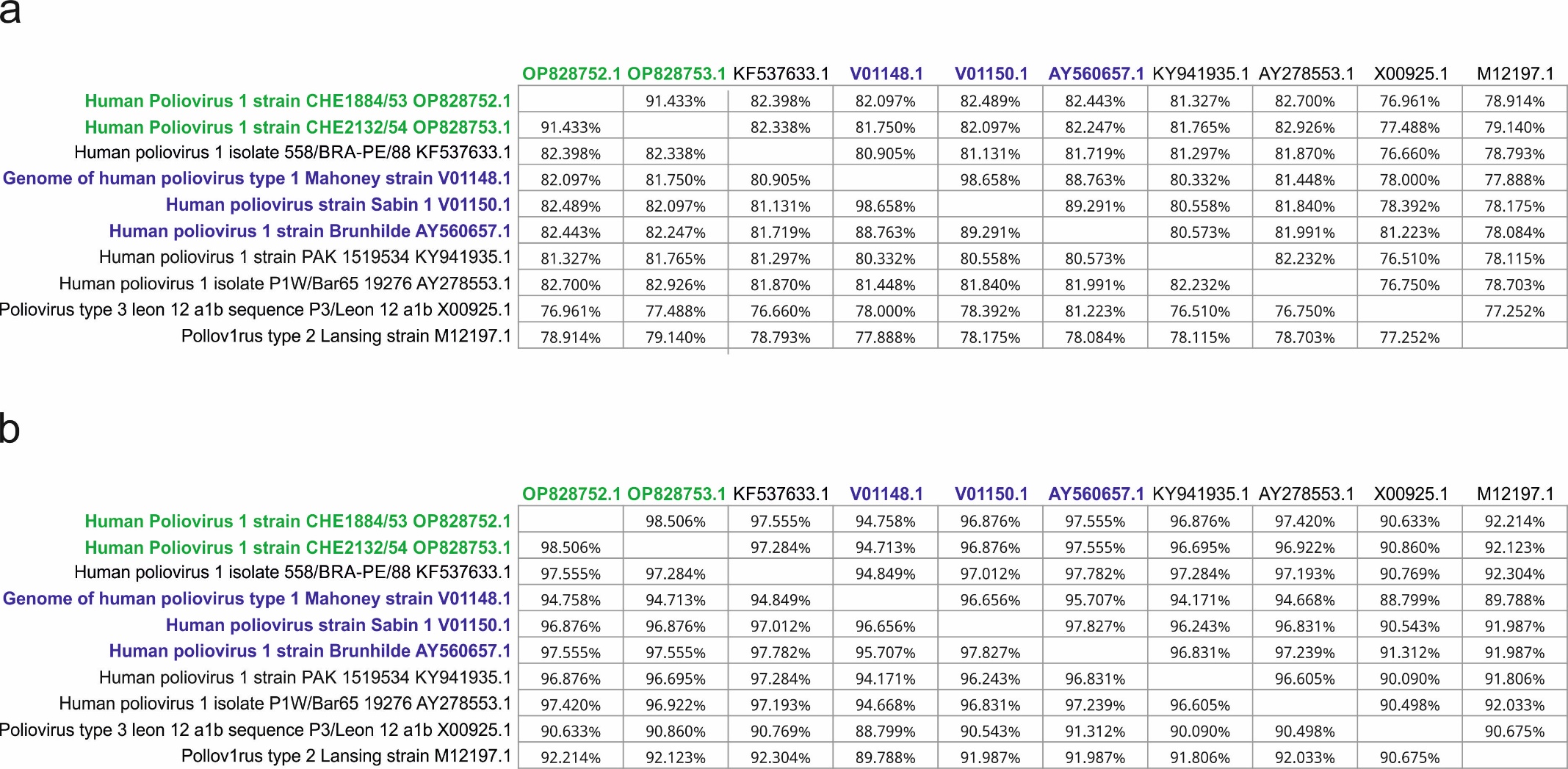


**Figures**
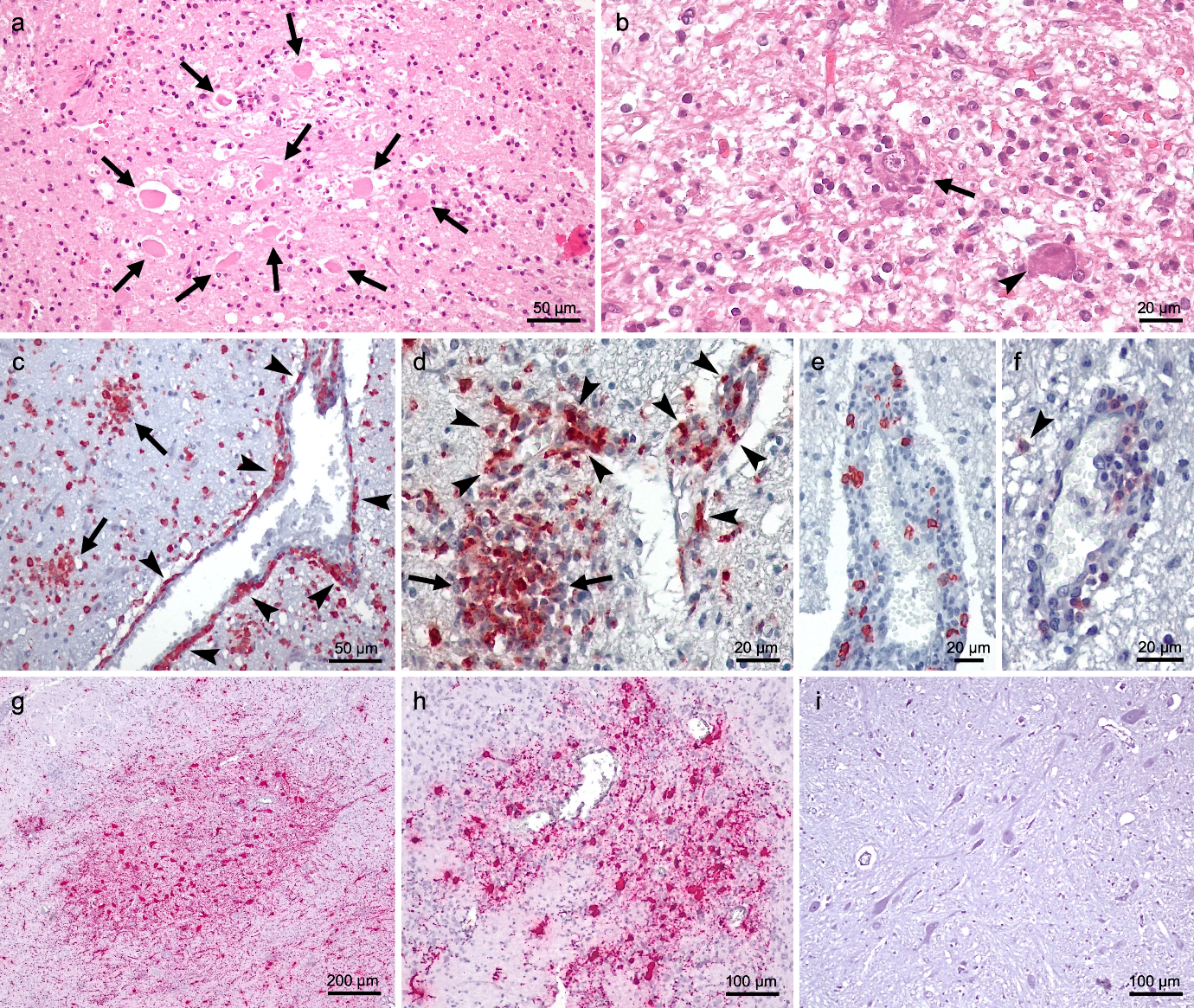


**Supplementary Fig. 1** a) Numerous axonal spheroids (arrows) in the anterior horn of the spinal cord of patient #2. b) Microglia/macrophages and fewer neutrophils surrounding and partially infiltrating a neuron (neuronophagia, arrow), and a necrotic neuron (arrowhead) in the spinal trigeminal nucleus of the medulla oblongata of patient #1. c & d) Immunohistochemistry for Iba1 (c) and neutrophil elastase (d) showing parenchymal clusters (arrows) and perivascular accumulation (arrowheads) of microglia/macrophages in the hypoglossal nucleus of the medulla oblongata (c) and of neutrophils in the anterior funiculus of the spinal cord (d) of patient #1. Neutrophils and microglia/macrophages were found in separate clusters. e & f) Immunolabeling of CD20-positive B- (e) and CD3-positive T- (f) lymphocytes inside perivascular cuffs in the superior cerebellar peduncle (e) and in perivascular cuffs and adjacent neuroparenchyma (f, arrowhead) of the lateral lemniscus (f) of the pons of patient #1. g & h) Detection of viral RNA in neurons and neuronal processes of the motor nucleus of the trigeminal nerve (g) and pontine nucleus (h) of patient #1. i) Neurons of the spinal trigeminal nucleus from the medulla oblongata of a patient without inflammatory lesions in the CNS tissue displaying no viral RNA (negative control for in-situ hybridization).

Magnification: a,c=20x; b,d-f=40x; g=4x; h,i=10x.


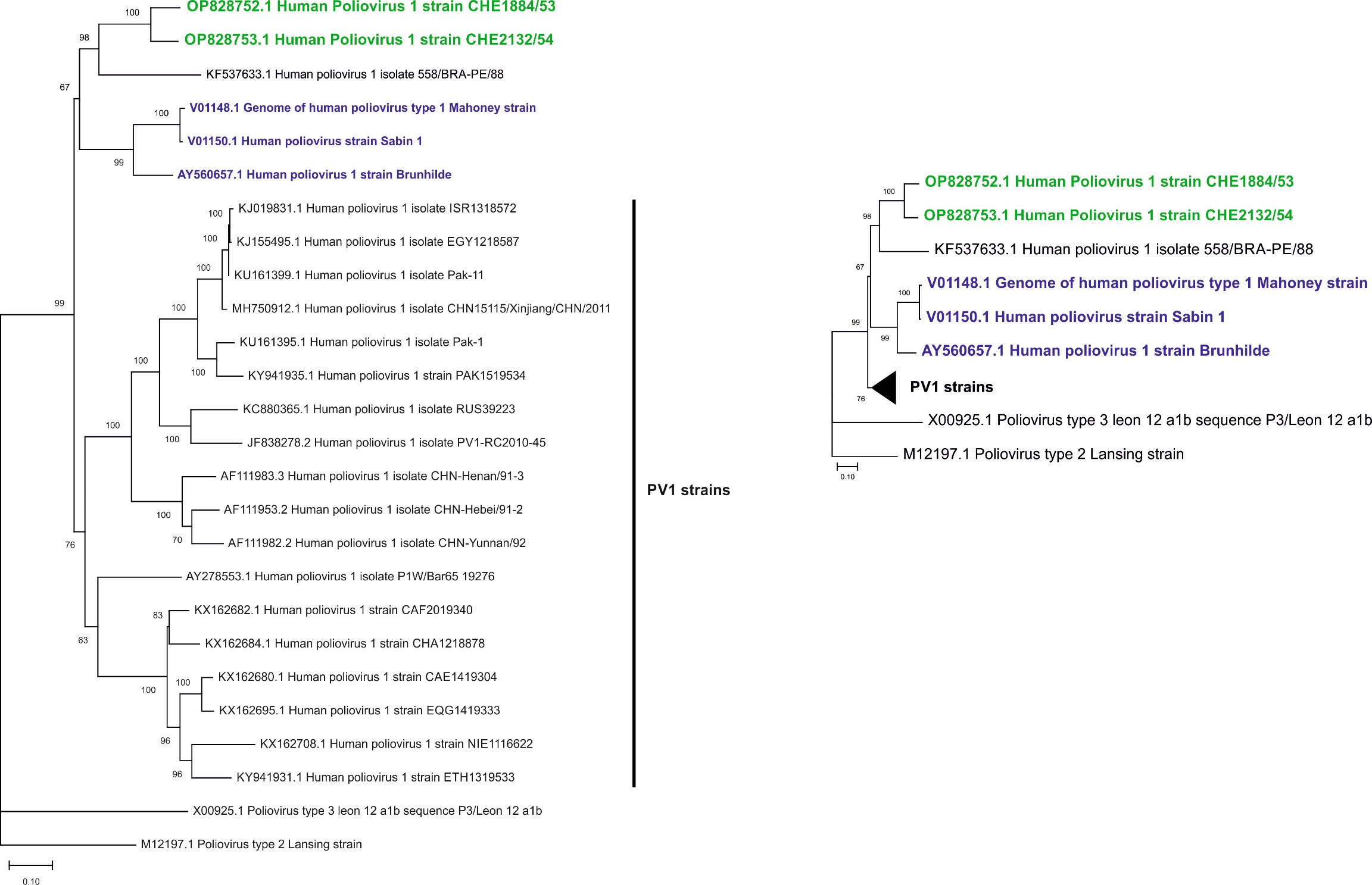


**Supplementary Fig. 2** Phylogenetic analysis of different full-length genomes of wild-type poliovirus 1. The analysis shows that only the Brazilian strain (KF537633), originally extracted from a fecal specimen [20], is closely related to our newly sequenced strains CHE2132/54 and CHE1884/53 (green). On the other hand, the PV1 wild-type sequences [5–7, 10, 16, 19–22] and three chimeric wild-vaccine PV1 strains (AF111953, AF111982, AF111983) [13] from sewage/feces specimen clustering in a different branch (two sequences are of unknown origin [21, 22]). The third branch represents the strains used for oral vaccine development (blue). Sequences of poliovirus type 2 (M12197) and type 3 (X00925) were used as outgroup. The alignment was performed with MAFFT [9] and the phylogenetic tree was constructed using MEGA X [12]. The graphics are assembled using CorelDraw X6 Version 16.0.0.707


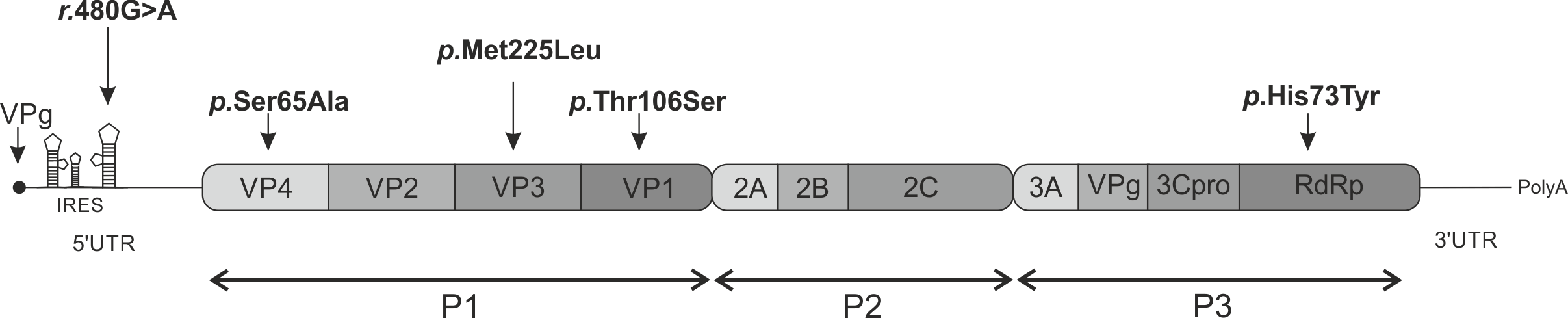


**Supplementary Fig. 3** Overview of the mutations within the poliovirus genome compared to the Sabin 1 strain. The substitutions are annotated according to their position in the polyprotein (composed of three precursor proteins P1 to P3), except the G>A substitution in the 5'UTR which is annotated according to the nucleotide position within the Mahoney strain. Despite the nucleotide change in the 5' UTR, our strains possessed the amino acid substitutions associated with neurotropism compared to the Sabin 1 strain in VP4 p.Ser65Ala, p.Met225Leu [18], and the p.His73Tyr substitution in the viral RNA polymerase gene leading to temperature-insensitivity of the poliovirus RNA polymerase in the uridylylation of VPg [17]. Moreover, other substitutions present in the Mahoney strain compared to the Sabin 1 strain, but differing in other neurovirulent strains (AY278553, AY560657, and KF537633) are in our strains as follows: in the VP1 p.Thr106Ala is instead p.Thr106Ser, and the p.Phe134Leu substitution is not present [18]. The graphics are assembled using CorelDraw X6 Version 16.0.0.707. Abbreviations.: VPg = viral protein linked to the genome; IRES = internal ribosomal entry site; UTR = untranslated region; VP 1-4 = viral protein 1-4; 3Cpro = 3C protease; RdRp = RNA dependent RNA poly

**References**

1. Buchfink B, Xie C, Huson DH (2015) fast and sensitive protein alignment using diamond. 12. doi: 10.1038/nmeth.3176

2. Camacho C, Coulouris G, Avagyan V, Ma N, Papadopoulos J, Bealer K, Madden TL (2009) BLAST+: Architecture and applications. BMC Bioinformatics 10:1–9. doi: 10.1186/1471-2105-10-421/FIGURES/4

3. Chen S, Zhou Y, Chen Y, Gu J (2018) fastp: an ultra-fast all-in-one FASTQ preprocessor. Bioinformatics 34:i884. doi: 10.1093/BIOINFORMATICS/BTY560

4. Dobin A, Davis CA, Schlesinger F, Drenkow J, Zaleski C, Jha S, Batut P, Chaisson M, Gingeras TR (2013) STAR: ultrafast universal RNA-seq aligner. Bioinformatics 29:15–21. doi: 10.1093/BIOINFORMATICS/BTS635

5. Drexler JF, Grard G, Lukashev AN, Kozlovskaya LI, Boẗtcher S, Uslu G, Reimerink J, Gmyl AP, Taty-Taty R, Lekana-Douki SE, Nkoghe D, Eis-Hübinger AM, Diedrich S, Koopmans M, Leroy EM, Drosten C (2014) Robustness against serum neutralization of a poliovirus type 1 from a lethal epidemic of poliomyelitis in the Republic of Congo in 2010. Proc Natl Acad Sci U S A 111:12889–12894. doi: 10.1073/PNAS.1323502111/-/DCSUPPLEMENTAL

6. Furtak V, Roivainen M, Mirochnichenko O, Zagorodnyaya T, Laassri M, Zaidi SZ, Rehman L, Alam MM, Chizhikov V, Chumakov K Environmental surveillance of viruses by tangential flow filtration and metagenomic reconstruction. 1. doi: 10.2807/1560-7917

7. Gerloff N, Sun H, Mandelbaum M, Maher C, Nix WA, Zaidi S, Shaukat S, Seakamela L, Nalavade UP, Sharma DK, Oberste MS, Vega E (2018) Diagnostic Assay Development for Poliovirus Eradication. J Clin Microbiol 56. doi: 10.1128/JCM.01624-17

8. Greenfield JG (2002) Greenfield’s Neuropathology. In: Graham DI, Lantos PL (eds) Viral diseases, 7th ed. Arnold, London, pp 10–15

9. Katoh K, Misawa K, Kuma KI, Miyata T (2002) MAFFT: a novel method for rapid multiple sequence alignment based on fast Fourier transform. Nucleic Acids Res 30:3059–3066. doi: 10.1093/NAR/GKF436

10. Korotkova EA, Park R, Cherkasova EA, Lipskaya GY, Chumakov KM, Feldman E V., Kew OM, Agol VI (2003) Retrospective Analysis of a Local Cessation of Vaccination against Poliomyelitis: a Possible Scenario for the Future. J Virol 77:12460. doi: 10.1128/JVI.77.23.12460-12465.2003

11. Küchler L, Rüfli I, Koch MC, Hierweger MM, Kauer R V., Boujon CL, Hilbe M, Oevermann A, Zanolari P, Seuberlich T, Gurtner C (2020) Astrovirus-Associated Polioencephalomyelitis in an Alpaca. Viruses 13. doi: 10.3390/v13010050

12. Kumar S, Stecher G, Li M, Knyaz C, Tamura K (2018) MEGA X: Molecular evolutionary genetics analysis across computing platforms. Mol Biol Evol 35:1547–1549. doi: 10.1093/molbev/msy096

13. Liu H-M, Zheng D-P, Zhang L-B, Oberste MS, Kew OM, Pallansch MA (2003) Serial Recombination during Circulation of Type 1 Wild-Vaccine Recombinant Polioviruses in China. J Virol 77:10994–11005. doi: 10.1128/JVI.77.20.10994-11005.2003

14. Nguyen LT, Schmidt HA, Von Haeseler A, Minh BQ (2015) IQ-TREE: a fast and effective stochastic algorithm for estimating maximum-likelihood phylogenies. Mol Biol Evol 32:268–274. doi: 10.1093/MOLBEV/MSU300

15. Nurk S, Bankevich A, Antipov D, Gurevich AA, Korobeynikov A, Lapidus A, Prjibelski AD, Pyshkin A, Sirotkin A, Sirotkin Y, Stepanauskas R, Clingenpeel SR, Woyke T, McLean JS, Lasken R, Tesler G, Alekseyev MA, Pevzner PA (2013) Assembling Single-Cell Genomes and Mini-Metagenomes From Chimeric MDA Products. J Comput Biol 20:714. doi: 10.1089/CMB.2013.0084

16. Omata T, Kohara M, Kuge S, Komatsu T, Abe S, Semler BL, Kameda,’ Heihachi Itoh A, Arita § Mineo, Wimmer E, Nomoto2 A (1986) Genetic Analysis of the Attenuation Phenotype of Poliovirus Type 1

17. Paul A V., Mugavero J, Yin J, Hobson S, Schultz S, Van Boom JH, Wimmer E (2000) Studies on the attenuation phenotype of polio vaccines: Poliovirus RNA polymerase derived from Sabin type 1 sequence is temperature sensitive in the uridylylation of VPg. Virology 272:72–84. doi: 10.1006/VIRO.2000.0354

18. Savolainen-Kopra C, Blomqvist S (2010) Mechanisms of genetic variation in polioviruses. Rev Med Virol 20:358–371. doi: 10.1002/RMV.663

19. Shulman LM, Gavrilin E, Jorba J, Martin J, Burns CC, Manor Y, Moran-Gilad J, Sofer D, Hindiyeh MY, Gamzu R, Mendelson E, Grotto I (2014) Molecular epidemiology of silent introduction and sustained transmission of wild poliovirus type 1, Israel, 2013. Euro Surveill 19. doi: 10.2807/1560-7917.ES2014.19.7.20709

20. Tavares FN, da Costa E V., Kew OM, da Silva EE (2013) Complete Genome Sequence of the Last Representative Genotype of Wild Indigenous Poliovirus Type 1, Which Circulated in Brazil. Genome Announc 1. doi: 10.1128/GENOMEA.00811-13

21. Human poliovirus 1 isolate RUS39223, complete genome - Nucleotide - NCBI. https://www.ncbi.nlm.nih.gov/nuccore/KC880365. Accessed 15 Sep 2022

22. Human poliovirus 1 isolate CHN15115/Xinjiang/CHN/2011, complete genome - Nucleotide - NCBI. https://www.ncbi.nlm.nih.gov/nuccore/MH750912. Accessed 15 Sep 2022
